# Unveiling Ga(III) phthalocyanine—a different photosensitizer in neuroblastoma cellular model

**DOI:** 10.1111/jcmm.14009

**Published:** 2018-11-19

**Authors:** Carolina Constantin, Andreea‐Roxana Lupu, Tudor Emanuel Fertig, Mihaela Gherghiceanu, Sevinci Pop, Rodica‐Mariana Ion, Monica Neagu

**Affiliations:** ^1^ Immunology Department “Victor Babes” National Institute of Pathology Bucharest Romania; ^2^ Pathology Department “Colentina” Clinical Hospital Bucharest Romania; ^3^ The Pathology Unit “Victor Babes” National Institute of Pathology Bucharest Romania; ^4^ Molecular and Cellular Medicine Department “Victor Babes” National Institute of Pathology Bucharest Romania; ^5^ Nanomedicine Research Group National Institute for Research & Development in Chemistry and Petrochemistry Bucharest Romania; ^6^ Doctoral School, Faculty of Biology University of Bucharest Bucharest Romania

**Keywords:** gallium(III), neuroblastoma, photodynamic therapy, photosensitizer, proliferation, toxicity, tumour, viability

## Abstract

Phthalocyanines (Pc) and their metallated derivatives are strongly considered for photodynamic therapy (PDT) possessing unique properties as possible new photosensitizers (PS). We have used toxicological assessments, real‐time monitoring of cellular impedance, and imagistic measurements for assessing the in vitro dark toxicity and PDT efficacy of Ga(III)‐Pc in SHSy5Y neuroblastoma cells. We have established the non‐toxic concentration range of Ga(III)‐Pc, a compound which shows a high intracellular accumulation, with perinuclear distribution in confocal microscopy. By choosing Ga(III)Pc non‐toxic dose, we performed in vitro experimental PDT hampering cellular proliferation. Our proposed Ga(III)‐Pc could complete a future PS panel for neuroblastoma alternate therapy.

## 1 INTRODUCTION

The current therapeutic lines for cancer treatment such as surgery, chemotherapy, and/or radiotherapy can cause significant trauma, systemic toxicity, and tissue injury, especially if repetitive treatment is necessary due to a refractory tumour. Therefore, the clinical interest in additional treatment strategies like photodynamic therapy (PDT) is steadily increasing in the last years. PDT is continuously expanding due to the efficient and selective photosensitizers (PS) accumulation that upon activation induces an increased tumour eradication. Moreover, this therapy has the advantage of being safely repeated when required.^1^ In PDT, a compound that has PS properties can be delivered by different routes (intravenously, intraperitoneally, or topically). PS is selectively accumulating in tumour cells, achieving a maximum accumulation in 3‐96 hours, depending on the PS nature and tumour type.^2^, ^3^ Conversely, sensitizer fluorescence could be used to diagnose and detect a certain tumour type. Alternatively, by irradiation at a specific wavelength, PS accumulated in the cell will generate reactive oxygen species responsible for apoptotic processes in tumour cells.^4^

Among different classes of PS, phthalocyanines (Pc) are currently intensely considered for PDT applications. Their metal derivatives are the most investigated chemical forms, taking into account both their photosensitizing/PS properties and imagistic applications. Pc and Pc derivatives are macrocyclic compounds easily activated by light at a specific wavelength.^5^ There are combined efforts focused to develop Pc forms for alternative cancer therapies, to decipher the mechanisms of action of Pc through PDT, its pharmaceutical development in order to enhance its “drugability,” and to improve its targeted intracellular distribution.^6^

Although gallium (Ga) has been discovered more than 100 years ago, its antitumour properties do not have a long historical record. Nevertheless, its potential to bind and modify some intracellular proteins, especially enzymes, has been exploited for tumour imaging for instance as ^67^Ga citrate.^7^, ^8^ Compounds with Ga and different chemical substituents (e.g., phenolate ligands or halogenated compounds) could induce apoptosis in C4‐2B prostate tumour cells or inhibit tumour development in mice‐bearing prostate cancer xenografts.^9^ Ga compounds may hold promise for antitumour treatment; however meanwhile, there is an utmost need to elucidate the mechanisms of Ga antitumor activity, type, and nature of its molecular targets in tumour cells.^10^ Furthermore, the cytotoxicity of Ga compounds and potential drug resistance represent significant challenges for biomedical scientists transposed in a continuous search for new Ga(III) formulas as therapeutic agents.^8^ Ga(III) salts are envisioned as therapeutical compounds because Ga(III) salts can be administrated orally being less toxic and allowing a chronic therapy. Moreover, this administration was shown that has an improved bioavailability for tumours compared to the parenteral use.^7^ In view of the anticancer potential of Ga(III) compounds, Pc‐containing Ga(III) could be a new type of PDT antineoplastic agent, expanding the Ga‐based therapeutic compounds list.^8^

Neuroblastoma is a solid tumour of the peripheral nervous system accounting for 8%‐10% of childhood tumours.^11^ Neuroblastoma is a tumour type in which Ga(III) compounds are used as possible imaging and therapy complexes.^12^ The current treatment for neuroblastoma involves a combination of surgery, radiation therapy, and chemotherapy (cisplatin and carboplatin) which unfortunately are prone to drug resistance development and toxic side effects. One way of overcoming chemotherapy side effects is to directly target the drug into the tumour and more so to track its delivery by in vivo imaging. Ga(III) could act as tumour imaging compound in radionuclide form or as part of chemical complexes for tumour therapy, having thus theranostic features.^13^, ^14^ A series of Ga(III) complexes comprising pyridine and phenol moieties encompassing the methoxy and halogenated groups was tested on a cisplatin‐resistant BE(2)‐C neuroblastoma cell line. The Ga(III)‐halogenated compounds induced apoptosis in BE(2)‐C neuroblastoma cells more efficiently than cisplatin and even superior to the nitro‐Ga(III) forms.^10^, ^12^

For PDT applications, Ga(III) fits better as PS in the form of Ga(III)Pc. It should be emphasized that Pc are of great interest as PS, by keeping the fine balance between the hydrophobic and hydrophilic characteristics of a qualified PS. Pc skeletons are mostly hydrophobic, but their solubility in the physiological milieu can be improved by chemical substitution, which increases its π‐electron density thus allowing solubilization in aqueous media. The chemistry of Pc is a promising area because there is a tremendous effort to search for water‐soluble forms (with inserted chemical groups such as sulphonates, carboxylates, phosphonates, and quaternary amino) and for controlling their tendency of aggregation in polar solvents. Furthermore, axial ligands included by coordination in complexes with Ga(III) prevents their molecular aggregation. In addition, Ga(III) could form PS with high triplet quantum yields and long triplet life‐times. Although attractive as PS, physico‐chemical studies with Ga(III)Pc are not abundant in the literature, and data related to cellular systems are even more rare. There are some studies, one of the first reports describing a tetra‐pyridyloxy cationic substituted water‐soluble Ga(III)PS as potential PDT agent, characterized by fluorescence and singlet oxygen release properties.^15^

In the light of the “theranostic” potential of Ga(III) complexes, our study focuses on testing a Ga(III)‐Pc [Ga(III)Pc] PS on SHSy5Y neuroblastoma cells in order to establish its toxicological safety for further PDT experimental therapies.

## 2 MATERIALS AND METHODS

Ga(III)‐Pc‐chloride was prepared according to a previously published methodology.^16^ Furthermore, the Ga‐compound was solubilized in dimethylsulphoxide (DMSO) in order to obtain a 1 mmol/L stock solution that was further diluted in cell medium to prepare all working solutions for cellular testing systems. The DMSO final concentration in the medium was <0.05% non‐toxic for cells.^17^ For irradiation PDT experiments, quartz cuvettes with 0.5‐2 cm optical path lengths containing 1 mL of SHSy5Y cell suspension each were used**.**

### 2.1 Cell line and treatments

Human neuroblastoma SH‐SY5Y adherent cell line with epithelial morphology (ATCC^®^ CRL‐2266™) was maintained in DMEM‐F12 modified medium (SAFC, Darmstadt, Germany) supplemented with 10% foetal bovine serum and 1% antibiotic‐antimycotic solution (Sigma‐Aldrich, Darmstadt, Germany). Cells were cultivated at 37°C in 5% CO_2_ atmosphere and subcultivated at 1:20 ratio using for cell detachment Trypsin‐EDTA 0.25% solution; the cells were maintained at 1 × 10^4^ cells per cm^2^ density. For dark cytotoxicity tests and experimental irradiation of Ga(III)Pc‐loaded cells, SH‐SY5Y were detached and counted by Trypan‐Blue exclusion test and further seeded in 96‐well plates at a density of 2 × 10^3^ per well. After 24 hours, cells were treated with different doses of Ga(III)Pc for 24, 48, and 72 hours. A stock solution of 1 mmol/L Ga(III)Pc was prepared in sterile conditions in DMSO and diluted accordingly in DMEM‐F12 modified medium.

### 2.2 Cellular viability

Lactate dehydrogenase (LDH) release assay was used for cellular viability and membrane integrity evaluation upon cells incubated with various doses of Ga(III)Pc. Cells were seeded at 2 × 10^3^ per well in 96‐well flat bottom plates and maintained at 37°C in humidified atmosphere with 5% CO_2_. After 24 hours, cells were treated with 0.4, 2, 10, and 20 μg/mL Ga(III)Pc.

Cytotox 96 Non‐Radioactive Cytotoxicity Assay kit (Promega, Madison, WI, USA) was used for assessing the released LDH from cells loaded with different doses of Ga(III)Pc as a viability test for both dark toxicity tests and PDT. For each type of sample, experiments were performed in triplicate and results were recorded spectrophotometrically and expressed as OD490 nm value compared to control.

### 2.3 Cellular proliferation

Cells were seeded at 2 × 10^3^ per well in 96 wells, maintained at 37°C in humidified atmosphere with 5% CO_2_, and after 24 hours, cells were treated with 0.4, 2, 10, and 20 μg/mL respectively Ga(III)Pc. The capacity of SH‐SY5Y cells loaded with Ga(III)Pc to proliferate was assessed as number of metabolically active cells with CellTiter96AQueous One Solution Cell Proliferation kit (Promega) for both dark toxicity tests and PDT. The test spectrophotometrically measures the intracellular dehydrogenases activity based on tetrazolium salt [3‐(4,5‐dimethylthiazol‐2‐yl)‐5‐(3‐carboxymethoxyphenyl)‐2‐(4‐sulfophenyl)‐2H‐tetrazolium, inner salt; MTS] reduction to a quantifiable formazan compound, reaction developed only by metabolically active cells.^18^ For each type of sample, three experiments were performed in triplicate. Results were expressed as OD490 nm value compared to untreated control cells.

### 2.4 Intracellular staining for confocal microscopy assessment

SHSy5Y cells were seeded at 5 × 10^4^ per cm^2^ cell density on 18 × 18 mm coverslips and cultured at 37°C in a humidified atmosphere with 5% CO_2_, prior to loading with Ga(III)Pc. After 24 hours, cells were exposed for 24 hours to 10 μg/mL Ga(III)Pc before dsDNA staining. Cells untreated with Ga(III)Pc were used as control. After Ga(III)Pc incubation, cells were fixed in 3.7% paraformaldehyde for 20 minutes, treated with glycine 20 mmol/L (3 × 5 minutes), permeabilizated 5 minutes with 0.1% Triton X, and incubated for 30 minutes with 1% bovine serum albumin (BSA). To help evaluate cell morphology, F‐actin was stained with Alexa Fluor‐488 phalloidin (Molecular Probes A 12379; Molecular Probes, Eugene, OR, USA) which was diluted 1:40 (BSA 1%) and applied to coverslips for 30 minutes; the DAPI solution was used for nuclei staining (10 minutes) and samples were mounted with Prolong Gold antifade reagent (Molecular Probes, P36934). All the solutions were prepared in PBS and the staining protocol was done at room temperature.

### 2.5 Confocal microscopy of cells loaded with Ga(III) compound

Confocal images were acquired using a Leica TCS SP8 system (Leica Microsystems, Wetzlar, Germany), equipped with an oil immersion objective (HC PL APO CS2 100x/1.40NA). A 405 nm UV laser and a white light laser set to 495 and 633 nm were used for the sequential excitation of fluorophores. Emission was registered with either a PMT detector (for DAPI) or HyD detectors (for Phalloidin‐488 and Ga(III)Pc). Scanning was done at a speed of 400 Hz and each line was averaged 6‐8 times. Image acquisition and 3D rendering of image stacks were performed using the manufacturer supplied LASX software (Leica Microsystems).

### 2.6 Experimental PDT

SH‐SY5Y cells were incubated for 24 hours in 10 μg/mL Ga(III)Pc and then washed twice in culture medium for the removal of the unbound compound. Trypsinized cell cultures were resuspended at 3 × 10^5^ cells per mL in culture medium and irradiated with a He‐Ne laser 5 mW, λ = 632.8 nm. Photoactivation was done for 30 minutes using an Hg lamp (375 nm with an interferential filter = 680 nm, at a distance of 30 cm; average irradiance 7 × 10^3^ J/m^2^.s) after a published method.^19^ A 600 nm glass cut‐off filter (SCHOTT AG, Mainz, Germany) and a water filter were used to remove ultraviolet and infrared radiations respectively. An interference filter (Intor, 670 nm with a band width of 40 nm) was additionally placed in the light path before the sample. Light intensities were measured with an incorporated detector (ICPE). After irradiation, cellular suspensions were washed twice in fresh culture medium for the removal of cell debris generated during irradiation and subjected to post‐irradiation testing.

### 2.7 Cellular impedance measurements

Impedance assessment was performed both for dark cytotoxicity and post‐PDT evaluation. The cellular impedance calculation provides a cell index value (CI) used to monitor cell viability, number, morphology, or adherence degree. It relies on analysing in real time the electrical impedance registered on gold microelectrodes arrayed on a grid in wells to which cells attach. As more cells are attaching on the microelectrodes, the electrode impedance is increasing.

For dark cytotoxicity, tests were run in four replicates on a RCTA‐DP (Roche Applied Science, Penzberg, Germany) by using E‐16 plates (ACEA Biosciences Inc., San Diego, CA, USA, cat. no. 05469830001). SHSY5Y cells were seeded at 2 × 10^3^ cells per well in E‐plates treated for 1 hour at 37°C with collagen I (Sigma C7661) 5 μg/mL in 0.1 mol/L NaHCO_3_ after an adapted protocol.^20^ To ensure a higher effect, we have used higher Ga(III)Pc doses compared with the previous tests. Cells in aliquots of 50 μL DMEM‐F12 medium were adhered in E‐16 plates for 2 hours at 37°C in 5% CO_2_ atmosphere, recording the impedance at every minute at RCTA‐DP unit. After 2 hours, Ga(III)Pc was added to adhered cells at 2, 10, and 50 μg/mL doses and impedance registered at every 1‐minute interval for a total period of 96 hours to monitor the proliferation of cells treated with Ga(III)Pc. The results were reported as normalized CI to the time before compound addition to the attached cells. The impedance results were automatically calculated as total CI registered at different time‐points.

For post‐irradiation assessment, cells loaded with 10 μg Ga(III)Pc were photoactivated and then cell proliferation was evaluated using impedance analysis at different time‐points post‐PDT. Thus, the CI index was registered at 2, 24, and 48 hours post‐PDT to evaluate if after irradiation the cells continue to proliferate. Impedance results are presented as automated CI registered for all tested time‐points.

### 2.8 Statistics

All the experiments were performed three times and for all the individual samples at least three replicates were done. Unless otherwise stated, all the data were presented as mean ± standard deviation; statistically significant was considered for *P* < 0.05. Proliferation indexes were calculated by dividing data for the tested sample to the data obtained for control. CI was automatically calculated by the Real‐Time Cell Analyzer Software.

## 3 RESULTS AND DISCUSSION

We aim to establish a toxicology pattern of a Ga(III)‐Pc as a potential therapeutical compound for antiproliferative effect in neuroblastoma cells. Thus, we assessed the effect of Ga(III)Pc on SHSy5Y cells, measuring viability, cellular proliferation, and real‐time cellular impedance, before and after the PDT experimental procedure. The entire workflow for assessing the dark toxicity profile was previously published by us in the approaches related to various (metallated) PS.^21^, ^22^, ^23^

### 3.1 Dark cytotoxicity of Ga(III)Pc in cellular system

**Figure 1:**
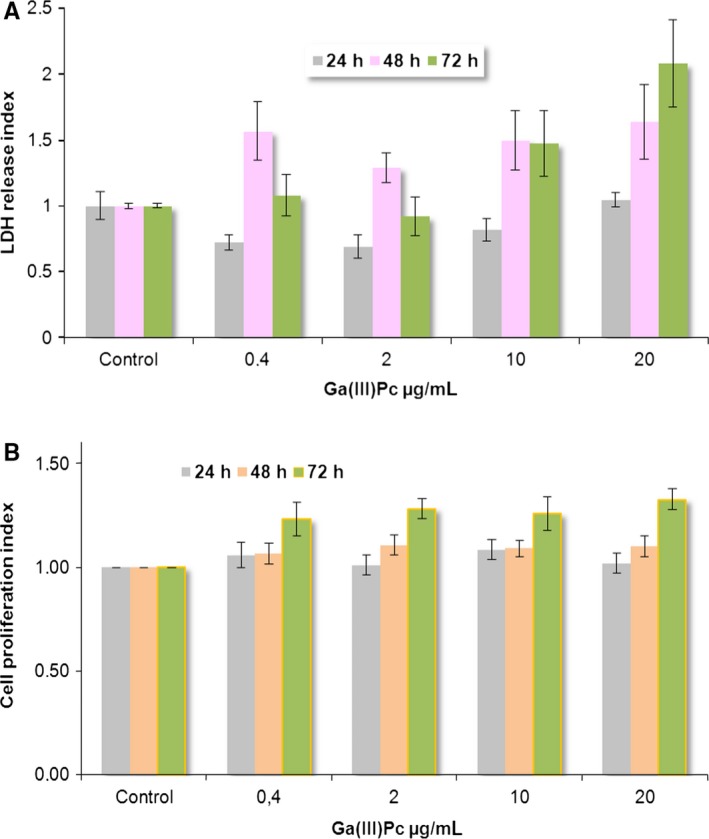
(A) Cellular viability assessed as lactate dehydrogenase (LDH) index for SHSy5Y cells incubated with Ga(III)Pc for different time‐points. (B) Cellular proliferation index for SHSy5Y cells incubated with Ga(III)Pc at different time‐points—assessed by MTS reduction in metabolically active cells

When assessing the viability of cells incubated with Ga(III)Pc at different time‐points, we have noticed that even at high concentration of compound (10 μg/mL), a low and relatively homogenous LDH index was obtained, especially after 24 hours of incubation in the compound; there were no statistical differences in LDH release at 24 hours for all tested doses. A modified pattern of LDH release was observed at prolonged cultivation; thus, at 48 hours, the index was higher at the lowest dose (0.4 μg/mL) then decreased following a similar pattern for higher doses. Interestingly, the cellular viability after 72‐hour incubation was preserved at the lowest doses and even better than those observed at 48 hours; after this point (2 μg/mL), the LDH index increased matching the decrease in cellular viability (Figure 1A).

Regarding cellular proliferation rate, it was noticed that for all tested Ga(III)Pc doses, the metabolic capacity of SHSy5Y cells was unaffected no matter the incubation time, the proliferation index displaying a similar pattern for all incubation times (Figure 1B). Interestingly, a slight increase in the proliferation index could be detected after 72 hours, but this did not correlate with LDH data at this time‐point where cellular viability started to decline.

**Figure 2:**
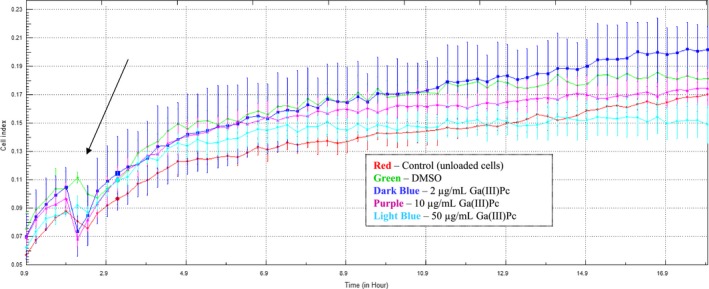
Cell index for SHSy5Y cells treated with Ga(III)Pc; 2000 cells/well; red = control (unloaded cells), green = DMSO, dark blue = 2 μg/mL GaPcCl; purple = 10 μg/mL Ga(III)Pc; light blue = 50 μg/mL Ga(III)Pc; black arrow detail—point when compound Ga(III)Pc is added to cells

As the tested cells are adherent and their cellular physiology resides on adherence, we cultivated cells in similar conditions and investigated impedance as a measure of their viability and adherence in real time, using an xCellIgence platform. At 2 hours from seeding in xCellIgence plates, the cellular adhesion was complete and the cell culture displayed a plateau CI. Thus, cells were monitored for the first 2 hours after seeding in the absence of compound; after the first 2 hours, different doses of Ga(III)Pc were added and cells were registered for 120 hours. Control cells (untreated with Ga(III)Pc) and cells with DMSO were run in parallel to subtract the potential effect of DMSO on cellular functionality in real‐time measurements. Investigating CI value in time, we observed that even the highest dose of Ga‐hindered cellular adherence only after prolonged incubation (>60 hours) (Figure 2). Neither DMSO nor the 50 μg dose influenced the CI and implicitly cellular behaviour in the time window related to dark cytotoxicity profile tests (72 hours). All the tested cellular systems, control cells, and cells incubated with lowest and medium Ga doses have a similar CI throughout the entire incubation time and have a similar pattern (Figure 2).

**Figure 3:**
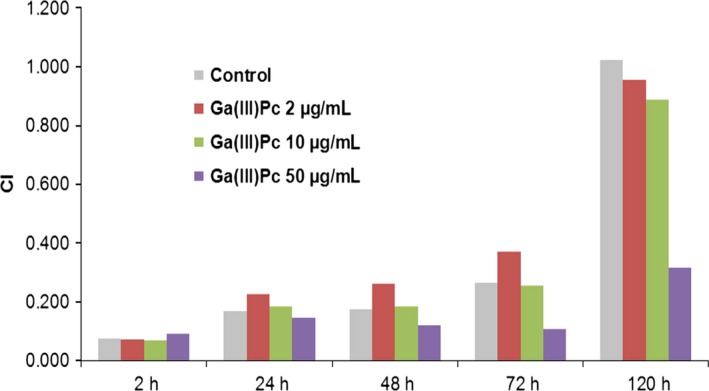
Cellular index (CI) for cellular impedance measurements—automated CI for 2, 24, 48, 72, and 120 h of SHSy5Y treated with various doses of Ga(III)Pc

With the exception of the highest tested dose (50 μg/mL), non‐irradiated Ga(III)Pc compound (“dark” experiments) did not hinder the proliferation capacity of the tested cells (Figure 3) and was further used in the experimental PDT.

Impedance measurements registered for Ga(III)Pc pointed out that CI changed depending on drug dose and was not influenced by the nature of chemical substituent of Pc.

Between impedance measurement analysis and MTS tests, there is a strong correlation showing that the tested concentrations do not hinder per se the cellular physiology.

### 3.2 Intracellular localization

**Figure 4:**
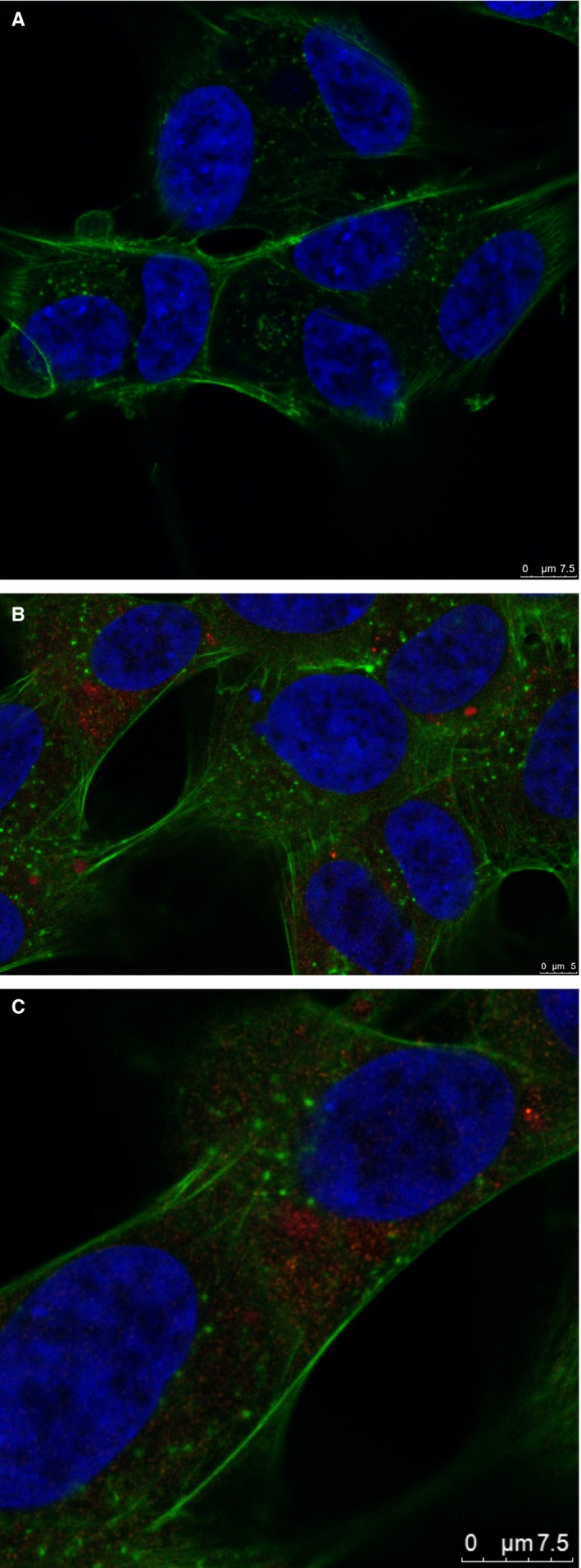
Confocal microscopy of SHSy5Y cells treated with Ga(III)Pc, showing the distribution of the compound. (A) Control—SHSy5Y cells without Ga(III)Pc, stained with phalloidin for F‐actin (green) and DAPI (blue). (B) SHSy5Y cells loaded with Ga(III)Pc (red) and stained with phalloidin (green) and DAPI (blue) show similar morphology to control cells. (C) Digital zoom of SHSy5Y cells loaded with Ga(III)Pc (red), showing a cytosolic, predominantely perinuclear distribution of the compound

In order to investigate the intracellular compartments in which PS accumulated and further reveal a possible apoptotic pathway, we used confocal microscopy to analyse cells loaded with Ga(III)Pc. There were no significant changes in morphology between control and treated cells, as revealed by phalloidin staining of F‐actin. PS showed a perinuclear/cytosolic distribution (Figure 4) and seemingly did not associate with cytoskeletal components. To confirm this distribution, in 3D, we generated an image stack which showed uniform accumulation of PS in the perinuclear cytoplasmic regions (supplementary file 3D rendering of image stack).

### 3.3 Experimental PDT

**Figure 5:**
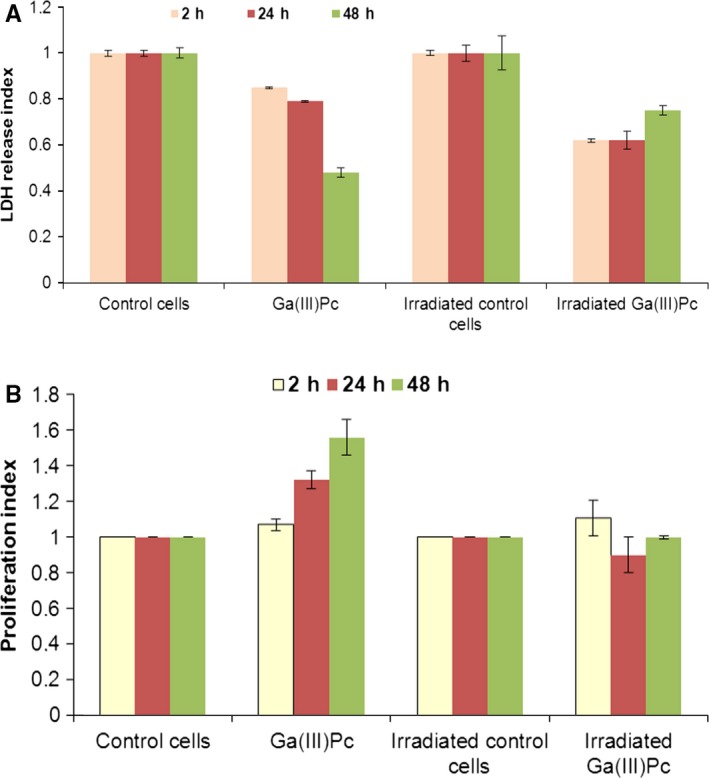
(A) Lactate dehydrogenase (LDH) release after experimental photodynamic therapy (PDT) of SHSy5Y loaded with 10 μg/mL Ga(III)Pc. (B) Proliferation index after experimental PDT of SHSy5Y loaded with 10 μg/mL Ga(III)Pc

Dark cytotoxicity data provided the optimal time incubation and concentration of Ga(III)Pc for experimental PDT. Thus, SHSY5Y cells were loaded with 10 μg/mL Ga(III)PC and incubated overnight and then subjected to PDT. After PDT photoactivation, cellular suspensions were washed twice in fresh culture medium for the removal of cell debris resulted at irradiation and subjected to post‐PDT analysis. A rapid post‐irradiation cell counts were performed in Trypan‐Blue exclusion test resulting a viability of 40% subsequently to PDT (data not shown). Thus, as a fraction of Ga(III)Pc cells are PDT‐resistant we examined cellular viability and proliferation at 2, 24 and 48 hrs post‐irradiation respectively. The resistant cells displayed a reduced LDH release compared to control cells for all investigated time‐points (Figure 5A). Moreover, especially at 2 hours post‐PDT, the proliferation index for Ga(III)Pc cells was slightly increased compared to unloaded irradiated SHSy5Y cells, a recovery suggesting that subtle molecular mechanisms are still to be elucidated. A number of factors could be involved in this “refractory” cellular behaviour to photo‐irradiation, such as cell morphology, apoptotic protein expression, or survival‐associated genes (Figure 5B).^24^

**Figure 6:**
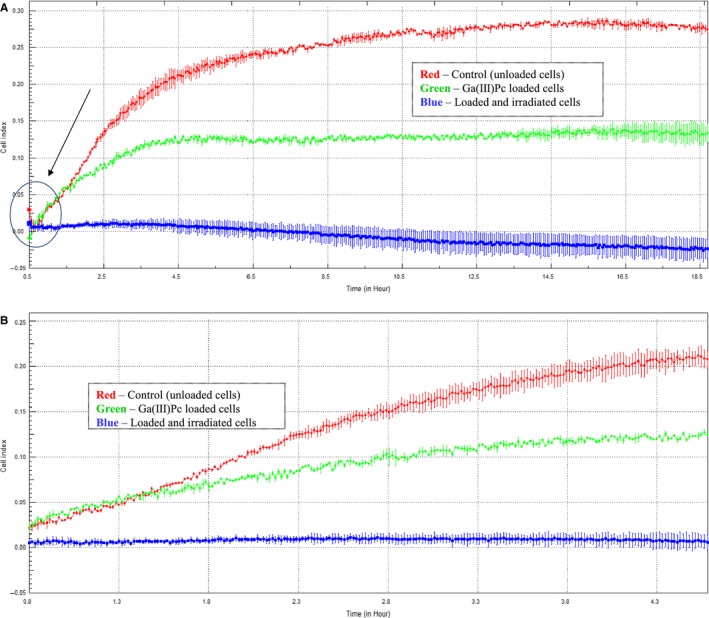
Impedance measurements results—cell index at first 24 h post‐PDT; (A) detail on first 18 h of monitoring; (B) detail on first 2 h of monitoring

**Figure 7:**
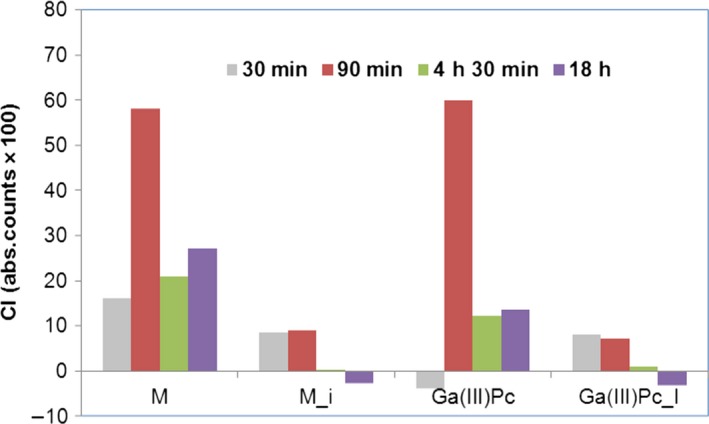
Cellular index of irradiated SHSy5Y cells loaded with Ga(III)Pc 10 μg/mL after different time periods cultivation post‐irradiation

In parallel with the viability and proliferation systems, we tested the cellular impedance of PDT‐resistant cells. Thus, SHSy5Y cells were seeded as in the dark toxicity evaluation and allowed for impedance monitoring for few days. It was observed at 1 hour from seeding, the CI of control and Ga(III)Pc non‐irradiated cells progress differently, a proper CI being noticed for control cells and, to a less extent, for non‐irradiated Ga(III)Pc‐loaded cells. The CI registered for Ga(III)Pc‐irradiated cells was maintained around Zero outline the entire period of monitoring. Interestingly, using LDH and MTS tests for PDT‐resistant cells, viability and proliferation could be registered, but CI registered after irradiation showed a constant decay of cellular adherence. This finding suggests that PDT finally took effect in cells (Figures 6A,B and 7).

Our experimental approach showed that this compound (Ga(III)Pc) can have new therapeutical valence as PS. Clinical data have shown that Ga(III) has a non‐specific accumulation and low tumour tissue penetrability.^25^ The Ga nitrate is clinically approved by FDA for the treatment of cancers such as lymphoma and bladder cancer. It is also approved for use in microbial infections, but its administration procedure is cumbersome and somewhat invasive.^8^ Therefore, finding a Ga(III) compound that can be directed towards inaccessible tumours, in non‐toxic concentrations for normal surrounding healthy tissues, and further activated using PDT procedures, is a clinical imperative. Ga(III) as Pc chloride represents an option, combining both its reported biomedical benefits with PDT procedure in order to induce cancer cell death. However, there are not many approaches with GaPc as an antitumour agent, while dark cytotoxicity profiles data are even less.

Our study tackles the toxicological portrait of Ga(III) inserted in Pc as potential PS agent used for experimental PDT upon SHSy5Y neuroblastoma cells. GaPc can be a good PS because it has good non‐toxic concentrations as proven by dark cytotoxicity tests. After PDT, SHSy5Y cells were killed in a high proportion (60%), however leaving a fraction of viable cells. This result comes in accordance with other reported data that show occasional cellular resistance to PDT, both for precancerous and malignant lesions,^24^ or with other studies that reveal certain genomic alterations linked to MAL‐PDT resistance in squamous cell carcinoma patients.^26^

As iron is known to be involved in cancer cell metabolism,^27^ Ga(III) compounds have also been used as an approach to perturb the iron metabolism of tumour cells by targeting key enzymes from iron homoeostasis pathways, especially iron‐dependent ribonucleotide reductase. One possible explanation of Ga (III) hindering cellular metabolism could rely on chemical properties similarities with Iron (III) and thus Ga(III) could enter in critical iron‐dependent cellular proliferation steps. Until now, the first generation compound Ga nitrate has demonstrated activity against bladder cancer and non‐Hodgkin's lymphoma in clinical trials. Further studies should be undertaken in order to identify additional biological targets of Ga(III) compounds in order to extend its clinical use in other tumour types.^28^ There is a fraction of PDT‐resistant cells that are not immediately killed by the photoactivated Pcs.^29^ We have shown that even this cell fraction can be affected by the PDT late action. Thus, we do not exclude two types of mechanisms, mutually probable. One is that cells that were not killed during PDT have suffered alterations that would be only detectable in the long term and the second explanation can be that although PS accumulated in the cells, the photosensitizer was not activated by PDT but induced metabolic hindrance, for example, interference with iron metabolism. Even at 4 days after PDT, the number of surviving cells did not reincrease and their proliferation/adherence capacity dramatically dropped, as compared to control cells.

Peripherally and non‐peripherally tetra‐substituted Zinc (II) Pcs bearing 2‐(2‐{2‐[3‐(dimethyl amino) phenoxy] ethoxy} ethoxy) ethoxy and 2‐(2‐{2‐[3‐(diethyl amino) phenoxy] ethoxy} ethoxy) ethoxy groups were synthesized along with their quaternary ionic derivatives and novel compounds were characterized by FT‐IR, (1)H NMR, (13)C NMR, UV‐vis, mass and elemental analyses assays. Owing to their excellent solubility in both organic and aqueous solutions, they are potential PS for use in PDT of cancer.^30^ The photophysical (fluorescence quantum yields and life‐times) and photochemical (singlet oxygen and photodegradation quantum yields) properties of these novel Pcs were exploited in light‐dependent photodamage in HeLa, HuH‐7,^31^ and K562 cancer cells.^32^ Using an established improved toxicological testing workflow,^33^ we found that the photosensitivity and the intensity of damage are directly related to the concentration of the PS. As brain tumours remain a major health concern with limited therapeutic options,^34^ our study has shown that neuroblastoma tumour cells can be damaged by PS, and in the future, these compounds may be attractive for clinical exploitation in both PDT and PDD.

In conclusion, in solid cancers, alternative treatment approaches like PDT are evolving in at least two directions, one by enlarging the tumour types where this can be applicable and second by designing new PS. Hence, our study has evaluated a new compound, Ga(III) Pc as a potential PS in neuroblastoma therapy. Our results have shown that Ga(III) Pc has a good toxicological profile and PS characteristics for applicable PDT in neuroblastoma cell line. In addition, our results point out new mechanisms that should be further investigated as the PS localization and the long‐term delayed decline effect post‐PDT indicate complex intracellular pathways triggering tumour cell death.

## CONFLICT OF INTEREST

The authors declare that there is no conflict of interest regarding the publication of this paper.
