# Supplementary figures and images for: Unveiling Ga(III) phthalocyanine—a different photosensitizer in neuroblastoma cellular model
Source: J Cell Mol Med. 2018 Nov 19;23(2):1086–94. doi: 10.1111/jcmm.14009 (PMC6349146; doi:10.1111/jcmm.14009)

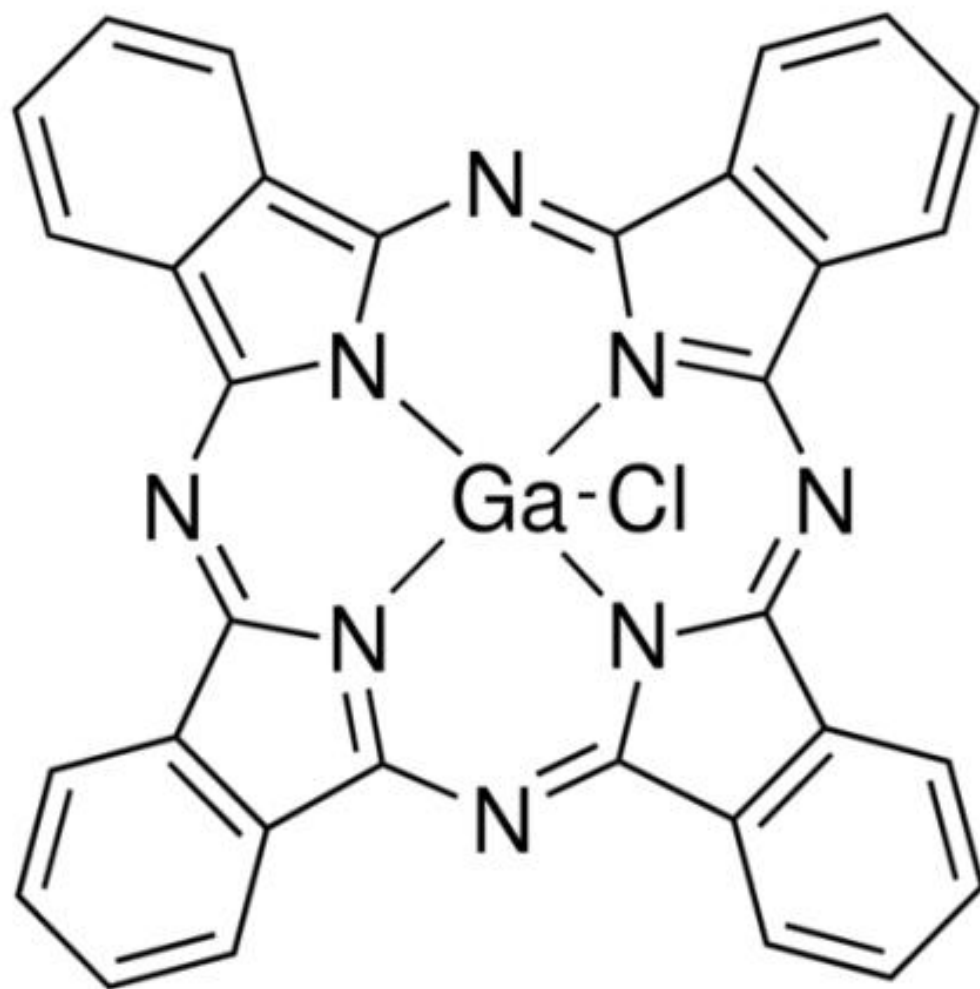

Supplement: Supplementary file 1 [file JCMM-23-1086-s001.pdf]
